# Adaptive Pathways of Microorganisms to Cope With the Shift From P- to N-Limitation in Subtropical Plantations

**DOI:** 10.3389/fmicb.2022.870667

**Published:** 2022-04-29

**Authors:** Chaoqun Wang, Ruzhen Jiao

**Affiliations:** ^1^Research Institute of Forestry, Chinese Academy of Forestry, Beijing, China; ^2^State Key Laboratory of Tree Genetics and Breeding, Chinese Academy of Forestry, Beijing, China; ^3^Key Laboratory of Tree Breeding and Cultivation of State Forestry Administration, Chinese Academy of Forestry, Beijing, China

**Keywords:** C:N:P ratio, stoichiometric imbalances, ecological stoichiometry, microbial community, enzymatic activity, microbial nutrient limitation

## Abstract

Ecological stoichiometry is increasingly acknowledged as one of the main control factors for microbial activity and diversity. Soil carbon/nitrogen (C/N) and carbon/phosphorus (C/P) ratios are usually much higher than microbial nutrient requirements and vary with planting density and stand age in forestlands. However, how microorganisms cope with such stoichiometric imbalances and how they regulate nutrient cycling remain unclear. Here, 5- and 35-year-old experimental *Cunninghamia lanceolata* [Lamb.] Hook plantations with five planting densities (1,667, 3,333, 5,000, 6,667, and 10,000 stems ha^−1^) were used to explore the underlying mechanism of the response of microorganisms to stoichiometric imbalances. We found that (i) enzyme activity and microbial biomass and diversity increased with planting density at age 5 but decreased at age 35; (ii) soil microorganisms were P-limited at age 5, but gradually shifted from P- to N-limitation during the development of plantations from 5 to 35 years; and (iii) significantly negative relationships between microbial biomass stoichiometry and enzymatic stoichiometry were observed. The adaptive pathways of soil microorganisms to cope with stoichiometric imbalances include (i) adjusting the stoichiometry of microorganisms and enzymes; (ii) changing the relative abundance of the dominant microbial phyla; and (iii) increasing the ratio of fungal to bacterial diversity. These results highlight how to use the ecological stoichiometry method to identify soil microbial nutrient limitations with planting density during the development of plantations. By underlining the important role of stoichiometry on microbial growth and activity, these findings furthermore emphasize the dependency of organic matter transformation and nutrient cycling on the microbial community.

## Introduction

Ecological stoichiometry theory can be used to predict how living organisms will absorb exterior environmental resources [usually carbon (C), nitrogen (N), and phosphorus (P)] at ecosystem scales (Hillebrand and Kahler, 2005; Marichal et al., 2011; Xu et al., 2017; Yang et al., 2020). For microorganisms, its biomass C/P and N/P ratios are commonly used as indicators of the bioavailability of P (Güsewell and Freeman, 2003; Tessier and Raynal, 2003). However, the stoichiometry of soil microbial biomass and its available C, N, and P are more restricted to a fairly narrow range at the global scale (Bell et al., 2013), and there are large differences between communities (Xu et al., 2013). Therefore, it is difficult to understand the non-equilibrium flow mechanism of ecosystem nutrients only by evaluating the stoichiometry of microbial biomass. Ecological enzyme (enzymes not contained by membranes of living cells) activity and their stoichiometry are indicators of microbial nutrient requirements since enzymes are regulated by the availability of nutrients (Sinsabaugh et al., 2009). For example, lower β-glucosidase/acid phosphatase ratio and β-1,4-N-acetylglucosaminidase/acid phosphatase ratio in forests indicate greater microbial P demand relative to P availability (Waring et al., 2014). Nonetheless, large uncertainties still exist due to the different responses of soil enzymatic activity and stoichiometry to nutrient stoichiometry (Cleveland and Liptzin, 2007; Burns et al., 2013). Therefore, studies on the stoichiometry of soil hydrolytic enzymes that are responsible for the mineralization of C, N, and P will provide deeper insights into the mechanism of soil organic matter transformation and nutrient cycling.

In forestlands, root exudates strongly affect the soil organic C availability and ultimately alter soil nutrient stoichiometry (Phillips et al., 2011). Moreover, soil C/N/P stoichiometry is closely related to the composition of understory vegetation, because the stoichiometry of terrestrial plant organs varies widely (Bell et al., 2013). For example, C/P and N/P of plant tissues vary in the range of 230–1,100 and 20–50, respectively (Bell et al., 2013). Therefore, changes in understory vegetation communities with plantation development will input organic matter sources with different C/N/P stoichiometry into the soil. This eventually causes imbalances in microbial nutrient requirements (Cleveland and Liptzin, 2007). Furthermore, root exudates and understory communities widely vary with planting density during the development of plantations due to the change in the intensity of competition for environmental resources such as light, water, and nutrients (Bremer and Farley, 2010). For instance, decreased planting density supported better maintenance of understory vegetation in *Pinus radiata* plantations (Brockerhoff et al., 2003). The total number of understory plant species, density, and diversity gradually increased in 5–10 years after the establishment of *Eucalyptus grandis* plantations (Zhang et al., 2014). Therefore, large uncertainties concerning the effects of planting density during the development of plantations on ecological stoichiometry are huge challenges for predicting soil nutrient cycling and sustainable development of afforestation ecosystems.

Various responses of soil bacterial and fungal community diversity and composition to stoichiometric imbalances following afforestation were observed in different plantations (Deng et al., 2016; Zhang et al., 2016; Ren et al., 2017; Wang et al., 2020). This is mainly because bacteria and fungi have different C/N/P stoichiometries and target to decompose organic matters according to the theory of ecological stoichiometry (Mooshammer et al., 2014). For example, fast-growing bacteria with lower C/P or N/P ratios mainly decompose labile and nutrient-rich organic matter to meet their elemental requirements (Ganie et al., 2016), whereas fungi are the main controllers of N and P mobilization rates from root exudates (Li et al., 2015). Therefore, the fungal-to-bacterial ratio can be considered an indicator of nutrient availability (Vries et al., 2006). Changes in soil C/N/P stoichiometries affect microbial community compositions, diversity, and functions (Elser et al., 2000; Aanderud et al., 2018). For instance, the N/P ratio is the predominant factor for the abundances of Proteobacteria, Bacteroidetes, Cyanobacteria, and Nitrospirae in *Robinia pseudoacacia* L. forests (Ren et al., 2017). The N addition strongly decreased microbial respiration in *Pinus koraiensis* plantation soils (Zhou et al., 2017). Despite recent improvements in understanding the underlying mechanisms for the response of the microbial community to afforestation, fundamental questions regarding how microorganisms cope with stoichiometric imbalance following afforestation are still unclear. Therefore, it is crucial to investigate microbial distribution and composition and the relationship among microorganisms and soil biotic and abiotic properties following afforestation.

In this study, we evaluated enzyme activities and microbial biomass, diversity, and composition with planting density in 5- and 35-year-old *Cunninghamia lanceolata* [Lamb.] Hook (Chinese fir) plantations, which account for 5% of all plantation areas worldwide (Ma et al., 2007). We examined the stoichiometry of soil, microbial biomass C (MBC), N (MBN), and P (MBP), and C (β-glucosidase, BG)-, N (leucine aminopeptidase, LAP, and β-1,4-N-acetylglucosaminidase, NAG)-, and P (acid phosphatase, AP)-acquiring enzymes to address two fundamental questions: How does planting density affect C/N/P stoichiometry with the development of plantations? And, how do microbial communities cope with stoichiometric imbalances? We hypothesized (i) that there may be stoichiometric imbalances between soil microorganisms and their demand for nutrients and that microorganisms would be restricted by P due to increasing depletion of P and competition intensity with planting density during the development from 5 to 35 years (Wang et al., 2021b); (ii) that microbial biomass and diversity would decrease with planting density due to increasing competitiveness of plants, and thus enzyme activity would also decrease; and (iii) that the stoichiometry of microbial biomass would negatively correlate with enzymatic stoichiometry since microorganisms optimize growth by allocating more reserves to acquire the most limited nutrient (Treseder and Vitousek, 2001).

## Materials and Methods

### Sampling Site and Experimental Design

This study was carried out in 5-year-old (5a) and 35-year-old (35a) Chinese fir plantations, located in Shanxi Forest Farm (27°44′ N, 114°39′ E) in Jiangxi Province and Weimin Forest Farm (27°05′ N, 117°43′ E) in Fujian Province, China, respectively (Supplementary Figure 1). The two study sites are located in the mid-subtropical region of China. The annual average temperature of the region is 16.8–19.4°C, and the annual average precipitation is 1,590–1,712 mm. The soil is typical Ferrosols developed from highly weathered granites (Huang et al., 2017). The site index, the average tree height of 20-year-old Chinese fir, of the two study sites was 16 and was recorded by the farms. This allows us to compare the differences in soil parameters of Chinese fir plantations between two stand ages.

The 5- and 35-year-old plantations were established with 1-year-old seedlings in a completely randomized block design with three blocks in 2013 and 1982, respectively. Each block consisted of five planting densities: 1,667 (D1), 3,333 (D2), 5,000 (D3), 6,667 (D4), and 10,000 stems ha^−1^ (D5), resulting in 15 plots at each age (30 plots in total). The area of each plot is 600 m^2^. The stand density, mean tree height, diameter at breast height (DBH), fine root biomass, and standing litter in each plot were measured in May 2017 (Table 1). The stand density of each plot is lower than the planting density because of the self-thinning rule rather than the artificially thinning.

**Table 1:** Characteristics of the plots of different planting densities in 5- and 35-year-old plantations.

	**5-Year-Old plantations**					**35-Year-Old plantations**				
	**D1**	**D2**	**D3**	**D4**	**D5**	**D1**	**D2**	**D3**	**D4**	**D5**
**Stand**										
Stand density (stem ha^−1^)	1,604 ± 20E	3,213 ± 16D	4,726 ± 21C	6,157 ± 56B	8,992 ± 69A	1,578 ± 96d	2,294 ± 38c	2,617 ± 223ab	2,789 ± 98a	2,461 ± 86bc
Mean diameter at breast height (cm)	4.9 ± 0.2A	4.7 ± 0.2AB	4.6 ± 0.3BC	4.3 ± 0.3BC	4.1 ± 0.1C	22.4 ± 2.4a	20.8 ± 1.1ab	18.7 ± 0.6b	16.3 ± 0.4c	15.8 ± 0.5c
Mean tree height (m)	5.8 ± 0.2A	5.7 ± 0.2A	5.5 ± 0.2A	5.5 ± 0.2A	5.1 ± 0.2B	22.6 ± 0.9a	21.9 ± 0.7b	19.6 ± 0.7c	18.8 ± 1.0c	18.6 ± 0.7c
Understory vegetation biomass (kg ha^−1^)	1,255 ± 64A	1,186 ± 123A	955 ± 33B	786 ± 15C	646 ± 84C	2,690 ± 290a	2,635 ± 175a	2,456 ± 50a	1,571 ± 161b	1,910 ± 133b
Fine root biomass (kg ha^−1^)	1,562 ± 112C	1,684 ± 132BC	1,790 ± 85B	1,902 ± 79B	2,263 ± 45A	4,279 ± 223c	5,408 ± 134b	6,816 ± 127a	7,111 ± 128a	7,009 ± 167a
Total litter stock	3,335 ± 214A	3,221 ± 208A	3,174 ± 80AB	2,819 ± 82BC	2,627 ± 188C	9,652 ± 546a	9,241 ± 795ab	8,227 ± 749ab	8,717 ± 826ab	7,679 ± 483b
**Soil**										
Soil moisture (%)	40.99 ± 0.62A	40.31 ± 0.35ABC	40.58 ± 0.27AB	39.48 ± 0.24C	39.74 ± 0.31BC	35.72 ± 2.31a	30.92 ± 2.62c	32.37 ± 1.49C	34.23 ± 0.58ab	32.61 ± 1.96bc
pH	4.70 ± 0.01B	4.74 ± 0.01A	4.72 ± 0.01AB	4.65 ± 0.01C	4.65 ± 0.01C	5.04 ± 0.02b	5.07 ± 0.02b	5.29 ± 0.01ab	5.49 ± 0.02a	5.27 ± 0.04ab
Organic carbon (g kg^−1^)	15.84 ± 0.96A	15.53 ± 0.59A	15.85 ± 0.16A	15.65 ± 0.37A	16.15 ± 0.57A	22.14 ± 2.17b	22.98 ± 1.14b	26.10 ± 1.26a	28.55 ± 1.04a	28.27 ± 0.86a
Total nitrogen (g kg^−1^)	1.45 ± 0.01C	1.60 ± 0.05B	1.25 ± 0.02D	1.06 ± 0.01E	1.91 ± 0.04A	1.61 ± 0.05a	1.56 ± 0.04a	1.55 ± 0.04a	1.57 ± 0.09a	1.52 ± 0.05a
Total phosphorus (mg kg^−1^)	368.53 ± 4.63A	364.67 ± 15.25A	364.93 ± 3.40A	355.20 ± 10.24A	356.10 ± 7.75A	289.77 ± 9.50a	260.50 ± 5.52bc	243.60 ± 6.83D	258.87 ± 3.17cd	275.83 ± 8.14ab
Microbial biomass carbon (mg kg^−1^)	480.67 ± 52.67B	493.67 ± 11.03B	583.83 ± 16.15A	604.50 ± 38.89A	588.00 ± 41.15A	1151.00 ± 73.73a	1204.40 ± 42.01a	901.10 ± 37.42b	679.80 ± 19.36c	707.87 ± 9.71c
Microbial biomass nitrogen (mg kg^−1^)	41.96 ± 2.43b	42.92 ± 5.69b	49.30 ± 2.78A	49.68 ± 2.78A	47.33 ± 2.02A	136.62 ± 1.57b	148.28 ± 2.12a	89.39 ± 0.86c	63.84 ± 1.27d	63.86 ± 1.27d
Microbial biomass phosphorus (mg kg^−1^)	4.43 ± 0.36C	4.91 ± 0.16C	5.86 ± 0.16B	6.63 ± 0.29A	6.11 ± 0.62AB	12.68 ± 0.24b	13.49 ± 0.33a	8.42 ± 0.12c	6.32 ± 0.08d	6.84 ± 0.11d

*The data are shown as the means ± standard deviations (n = 3). Different uppercase and lowercase letters represent significant differences between planting densities at age 5 and 35, respectively. D1, 1,667 stems ha^−1^; D2, 3,333 stems ha^−1^; D3, 5,000 stems ha^−1^; D4, 6,667 stems ha^−1^; D5, 10,000 stems ha^−1^*.

### Soil Sampling

In May 2017, eight soil samples with a diameter of 5 cm were collected from 0 to 20 cm by the “S-sampling” method (Li, 2016) in each plot, combined, sieved through a 2-mm mesh, air-dried, and divided into three subsamples. One subsample was stored at −80°C before the analysis of DNA; one was kept at 4°C for the analysis of soil microbial biomass carbon (MBC), nitrogen (MBN), phosphorus (MBP), the activities of β-glucosidase (BG), leucine aminopeptidase (LAP), β-1,4-N-acetylglucosaminidase (NAG), and acid phosphatase (AP) and another was air-dried and ground to measure soil organic carbon (SOC), total nitrogen (TN), and total phosphorus (TP).

### Soil Physicochemical Analyses and Microbial Biomass Assays

Soil moisture (SM) was measured by oven-drying samples at 105°C. Soil pH was measured in soil/water suspension (1:5). The SOC and TN contents were measured using the K_2_Cr_2_O_7_-H_2_SO_4_ oxidation method (Lefroy et al., 1993) and the Kjeldahl method (Bremner and Mulvaney, 1982), respectively. The TP content was measured after digestion with ammonium persulfate and sulfuric acid in an autoclave at 121°C on an ICP (Spectro Analytical Instruments, Spectro Arcos ICP, Kleve, Germany).

The MBC, MBN, and MBP contents were measured using the fumigation–extraction method (Brookes et al., 1984; Vance et al., 1987). The MBC and MBN contents were calculated according to the equations MBC = E_C_/k_EC_ and MBN = E_N_/k_EN_, where k_EC_ and k_EN_ are equal to 0.45 and E_C_ and E_N_ are the difference between the content of organic C and N extracted from the fumigated and non-fumigated soils, respectively. The MBP content was calculated using a conversion factor of 0.40 and the recovery of an inorganic P spike by measuring the difference between the content of extractable P extracted from the fumigated and non-fumigated soils.

### Soil Enzyme Activities Assays

The BG and AP activities were assayed using a substrate of pNPG (4-nitrophenyl-β-D-glucopyranoside) (Geng et al., 2012) and p-nitrophenyl phosphate (Tabatabai, 1994), respectively. Further details of the procedure for BG and AP assays can be found in Wang et al. (2021a,b). The activities of LAP and NAG were measured by the method of Saiya-Cork et al. (2002). In brief, 1 g of soil was added to 9 ml of 50 mM sodium acetate buffer. The mixed solution was shaken well and then centrifuged at 4°C for 20 min (2,000 rpm). Finally, the supernatant was collected. Then, 10 μl of the supernatant was distributed to each well of a 96-well black microplate, and the reagents were added according to the kit instructions (Qingdao Jieshikang Biotechnology Co., Ltd., Qingdao, China). Microplates were incubated at 37°C for 60 min. Fluorescence at 450 nm was determined on a SpectraMax Paradigm Multi-Mode detection platform (Molecular Devices, San Jose, CA, USA). Soil C/N, C/P, and N/P acquisition ratios were calculated using BG/(LAP+NAG), BG/AP, and (LAP+NAG)/AP, respectively.

### DNA Extraction, PCR Amplification, and Illumina Sequencing

The total soil microbial genomic DNA was extracted by following the manufacturer's protocols of the Power Soil DNA Isolation Kit (MoBio Laboratories, Carlsbad, CA, USA). The purity and quality of the extracted genomic DNA were checked by electrophoresis on 1% agarose gels. The PCR amplification of the bacterial V3–V4 region was conducted by using primers 338F (5′-GTACTCCTACGGGAGGCAGCA-3′) and 806R (5′-GTGGACTACHVGGGTWTCTAAT-3′) (Zhang et al., 2015). The 5′ end of the forward and reverse primers each contained a 10-digit barcode sequence (provided by Allwegene Company, Beijing, China). The PCRs were carried out in 25 μl reaction volumes with 12.5 μl KAPA 2G Robust Hot Start Ready Mix, 1 μl forward primer (5 μM), 1 μl reverse primer (5 μM), 5 μl template DNA (30 ng in quality), and 5.5 μl H_2_O. Cycling parameters were 95°C for 5 min, followed by 28 cycles of 95°C for 45 s, 55°C for 50 s, and 72°C for 45 s with a final extension at 72°C for 10 min. Three PCR products per sample were pooled to mitigate reaction-level PCR biases. The fungal ITS-1 region was amplified by using primers ITS1F (5′-GGAAGTAAAAGTCGTAACAAGG-3′) and ITS2 (5′-ATCCTCCGCTTATTGATATGC-3′) (White et al., 1990). The PCRs were conducted as follows: 4 μl 5 × FastPfu buffer, 1 μl forward primer (5 μM), 1 μl reverse primer (5 μM), 2 μl dNTP mixture (2.5 mM), 5 μl template DNA (30 ng in quality), and 10 μl H_2_O. Cycling parameters were 95°C for 2 min, followed by 30 cycles of 95°C for 30 s, 55°C for 30 s, and 72°C for 30 s with a final extension at 72°C for 5 min. Three separate PCR products per sample were pooled to mitigate reaction-level PCR biases.

The PCR products were purified using a QIAquick Gel Extraction Kit (QIAGEN, Germany), quantified by a real-time PCR, and sequenced on an Illumina MiSeq PE300 platform at Beijing Allwegene Tech, Ltd (Beijing, China). After the run, image analysis, base calling, and error estimation were performed using Illumina Analysis Pipeline Version 2.6 (Zhao et al., 2017). The erroneous and chimeric sequences were filtered out, and pair-end reads were initially trimmed using Illumina Analysis Pipeline Version 2.6 to remove low-quality sequences with a score below 20. Then, high-quality sequences were aligned to the Ribosomal Database Project (Cole et al., 2009) using QIIME (V1.7.0) (Caporaso et al., 2010) and were clustered into operational taxonomic units (OTUs) with a 97% similarity level using Uclust (Edgar, 2010). The taxonomy of each 16S rRNA and ITS gene sequence was analyzed by UCLUST against the Silva119 16S rRNA database using a confidence threshold of 90%. The initial sequencing data were submitted to the NCBI Sequence Read Archive (SRA) with the Bioproject of PRJNA811461. The Chao1 and Shannon diversity indexes were calculated using the QIIME in Python (v.1.8.0) (Sanner, 1999).

### Statistical Analysis

A one-way ANOVA, followed by the Tukey's HSD *post-hoc* test, was conducted to test the differences in enzyme activity, bacterial and fungal sequence and OUT numbers, microbial alpha diversity, the relative abundance of dominant microbial phyla, and soil, microbial, and enzymatic C/N/P stoichiometry among planting densities. The unary linear regression was conducted to identify the relationships among stoichiometry of soil, microbial, and enzymatic components. All statistical tests were conducted using SPSS (version 21, IBM, Armonk, NY, USA). Spearman's correlation analysis was performed to evaluate the relationships between enzyme activity and microbial alpha diversity (Chao1 and Shannon) and between microbial dominant phyla abundances and enzyme activities and soil physicochemical properties *via* one-way ANOVA in R (v.4.0.4). Redundancy analysis (RDA) was conducted to test the relative importance of soil physicochemical factors, microbial biomass C, N, and P, and soil and microbial C/N/P stoichiometry in explaining variations in microbial diversity using the “vegan” package in R. To eliminate multicollinearity, we selected explanatory variables using the variance inflation factor (VIF) lower than 10 (Tamura et al., 2019). Statistical analyses were tested using PERMANOVA based on 999 permutations in R.

## Results

### Soil Microbial Biomass and Enzyme Activities

**Figure 1:**
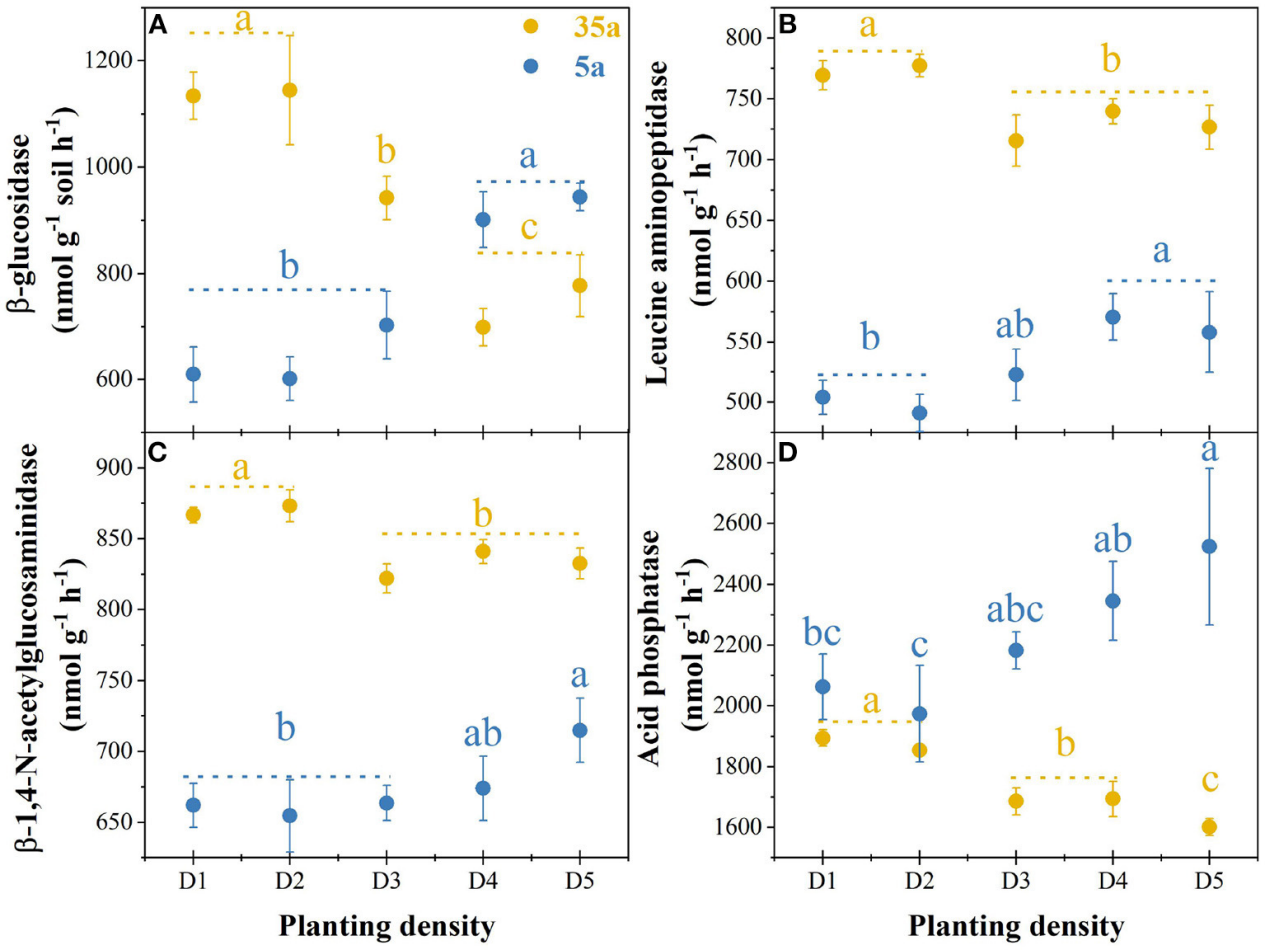
Enzyme activities in 5-year-old (5a, blue) and 35-year-old (35a, yellow) plantations. Different lowercase letters represent significant differences between different planting densities (*p* < 0.05). The data are shown as the mean ± standard deviations (*n* = 3). D1, 1,667 stems ha^−1^; D2, 3,333 stems ha^−1^; D3, 5,000 stems ha^−1^; D4, 6,667 stems ha^−1^; D5, 10,000 stems ha^−1^.

At age 5, MBC, MBN, and MBP in high planting densities (>3,333 stem ha^−1^, in D3, D4, and D5) were higher by 10% to 50% than in low planting densities (<3,333 stem ha^−1^, in D1–D2) (Table 1). However, the MBC, MBN, and MBP were lower by 22 to 57% in high vs. low planting densities at age 35 (Table 1). The activities of four tested enzymes increased with planting density at age 5, while the opposite pattern was observed at age 35 (Figure 1).

### Stoichiometry of Soil Nutrients, Microbial Biomass, and Enzyme Activities

**Figure 2:**
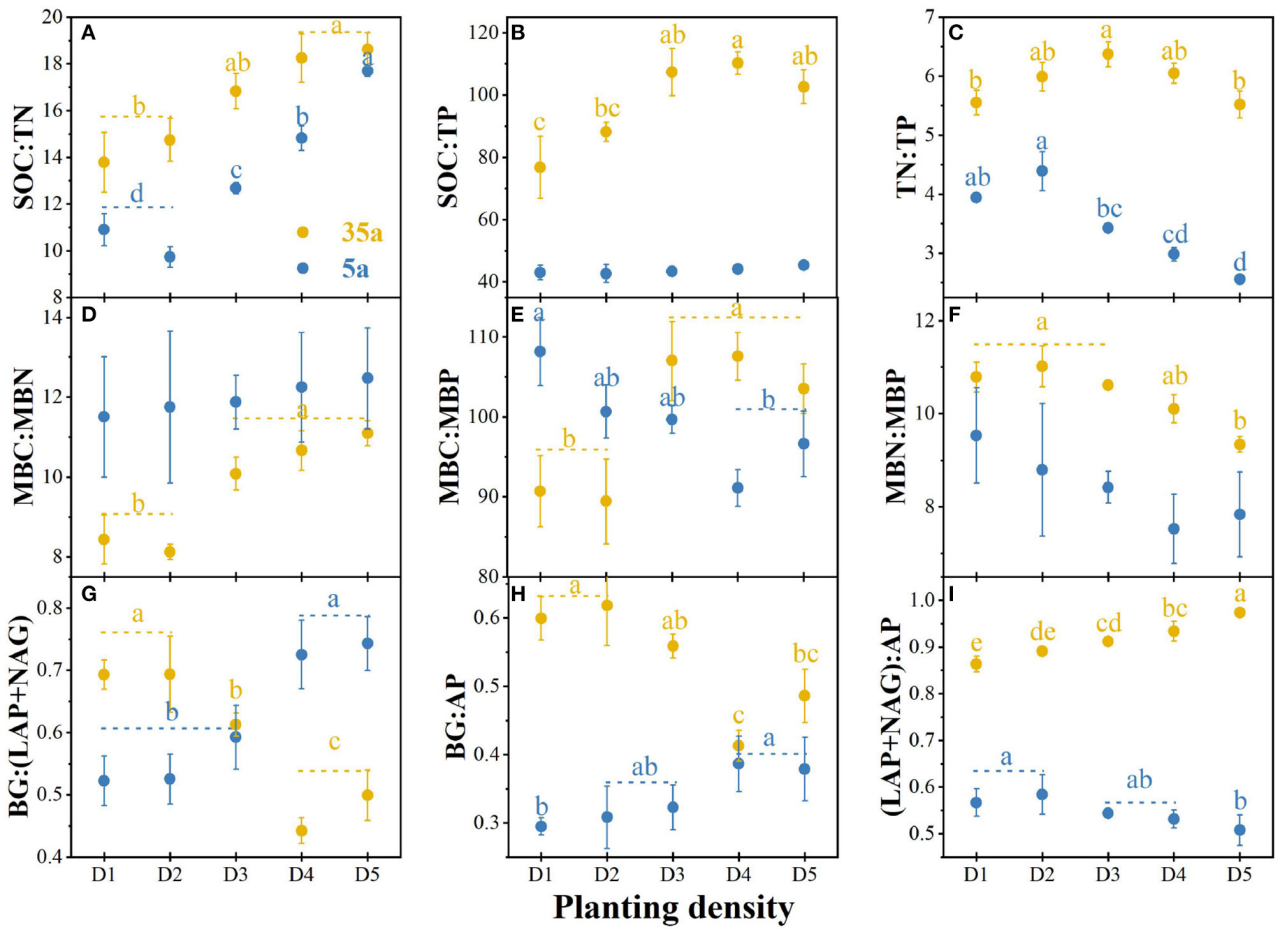
Soil carbon (C)/nitrogen (N)/phosphorus (P) stoichiometry, microbial biomass C/N/P stoichiometry, and enzymatic C/N/P stoichiometry in 5-year-old (5a, blue) and 35-year-old (35a, yellow) plantations. C acquisition is represented by β-glucosidase (BG); N acquisition is measured by the potential activities of leucine aminopeptidase (LAP) and β-1,4-N-acetylglucosaminidase (NAG); P acquisition is measured as acid phosphatase (AP) activity; MBC, microbial biomass carbon; MBN, microbial biomass nitrogen; MBP, microbial biomass phosphorus; SM, soil moisture; SOC, soil organic carbon; TN, total nitrogen; TP, total phosphorus. Different lowercase letters represent significant differences between different planting densities (*p* < 0.05). The data are shown as the mean ± standard deviations (*n* = 3). D1, 1,667 stems ha^−1^; D2, 3,333 stems ha^−1^; D3, 5,000 stems ha^−1^; D4, 6,667 stems ha^−1^; D5, 10,000 stems ha^−1^.

The SOC/TN ratio was higher in high vs. low planting densities at ages 5 and 35 (Figure 2A). The TN/TP ratio was higher in D1 and D2 than in D4 and D5 at age 5 (Figure 2C). The TN/TP ratio had the highest value in D3 and the lowest ones in D1 and D5 at age 35 (Figure 2C). At age 35, the SOC/TP ratio was higher in high vs. low planting densities (Figure 2B). The MBC/MBN ratio slightly increased with planting density at age 5, while MBC/MBP and MBN/MBP ratios were higher in D1, D2, and D3 vs. D4 and D5 (Figures 2D–F). At age 35, MBC/MBN and MBC/MBP ratios were higher in high vs. low planting densities, while MBN/MBP ratio was the opposite (Figures 2D–F). Finally, the opposite patterns for enzymatic C/N/P at ages 5 and 35 were observed (Figures 2G–I). At age 5, enzymatic C/N and C/P increased with planting density, while enzymatic N/P ratio decreased (Figures 2G–I).

### Soil Microbial Diversity and Composition

There were no differences in bacterial OTU number and Chao1 index among planting densities either in 5a or in 35a (Table 2, Supplementary Table 1). Fungal Shannon index and the ratio of fungal to bacterial Shannon increased with planting density at age 5 (Table 2). At age 35, fungal Chao1 and Shannon indexes decreased with planting density; moreover, the fungal-to-bacterial ratios of either Chao1 or Shannon index were higher in high vs. low planting densities (Table 2).

**Table 2:** Richness and diversity indices of bacterial (B) and fungal communities (F) in 5-year-old (5a) and 35-year-old (35a) Chinese fir plantations of different stand densities.

		**Chao1**			**Shannon**		
		**B**	**F**	**F/B**	**B**	**F**	**F/B**
5a	D1	3,429 ± 408a	536 ± 147a	0.15 ± 0.03a	9.29 ± 0.54a	5.80 ± 0.11c	0.63 ± 0.03b
	D2	3,570 ± 235a	583 ± 94a	0.17 ± 0.04a	9.56 ± 0.12a	6.17 ± 0.27bc	0.65 ± 0.02b
	D3	3,668 ± 122a	608 ± 79a	0.17 ± 0.02a	9.54 ± 0.29a	6.35 ± 0.16bc	0.67 ± 0.02ab
	D4	3,800 ± 266a	663 ± 121a	0.17 ± 0.02a	9.47 ± 0.45a	6.38 ± 0.44ab	0.67 ± 0.01ab
	D5	3,797 ± 186a	732 ± 117a	0.19 ± 0.02a	9.69 ± 0.17a	6.90 ± 0.11a	0.71 ±0.01a
35a	D1	1,405 ± 56a	1,459 ± 75a	0.96 ± 0.04b	7.77 ± 0.15a	6.50 ± 0.48a	1.20 ± 0.07b
	D2	1,468 ± 36a	1,448 ± 110a	1.02 ± 0.06b	7.90 ± 0.24a	6.57 ± 0.73a	1.20 ± 0.13b
	D3	1,371 ± 64a	1,314 ± 56ab	1.04 ± 0.06ab	7.71 ± 0.49a	5.72 ± 0.03b	1.35 ± 0.09ab
	D4	1,346 ± 139a	1,224 ± 91b	1.10 ± 0.04a	7.67 ± 0.34a	5.44 ± 0.29b	1.41 ± 0.04a
	D5	1,321 ± 163a	1,309 ± 106ab	1.01 ± 0.04b	7.42 ± 0.20a	5.90 ± 0.31 ab	1.26 ± 0.09ab

*The data are shown as the means ± standard deviations (n = 3). Different lowercase letters represent significant differences between planting densities in the same age plantations (p < 0.05). D1, 1,667 stems ha^−1^; D2, 3,333 stems ha^−1^; D3, 5,000 stems ha^−1^; D4, 6,667 stems ha^−1^; D5, 10,000 stems ha^−1^*.

**Figure 3:**
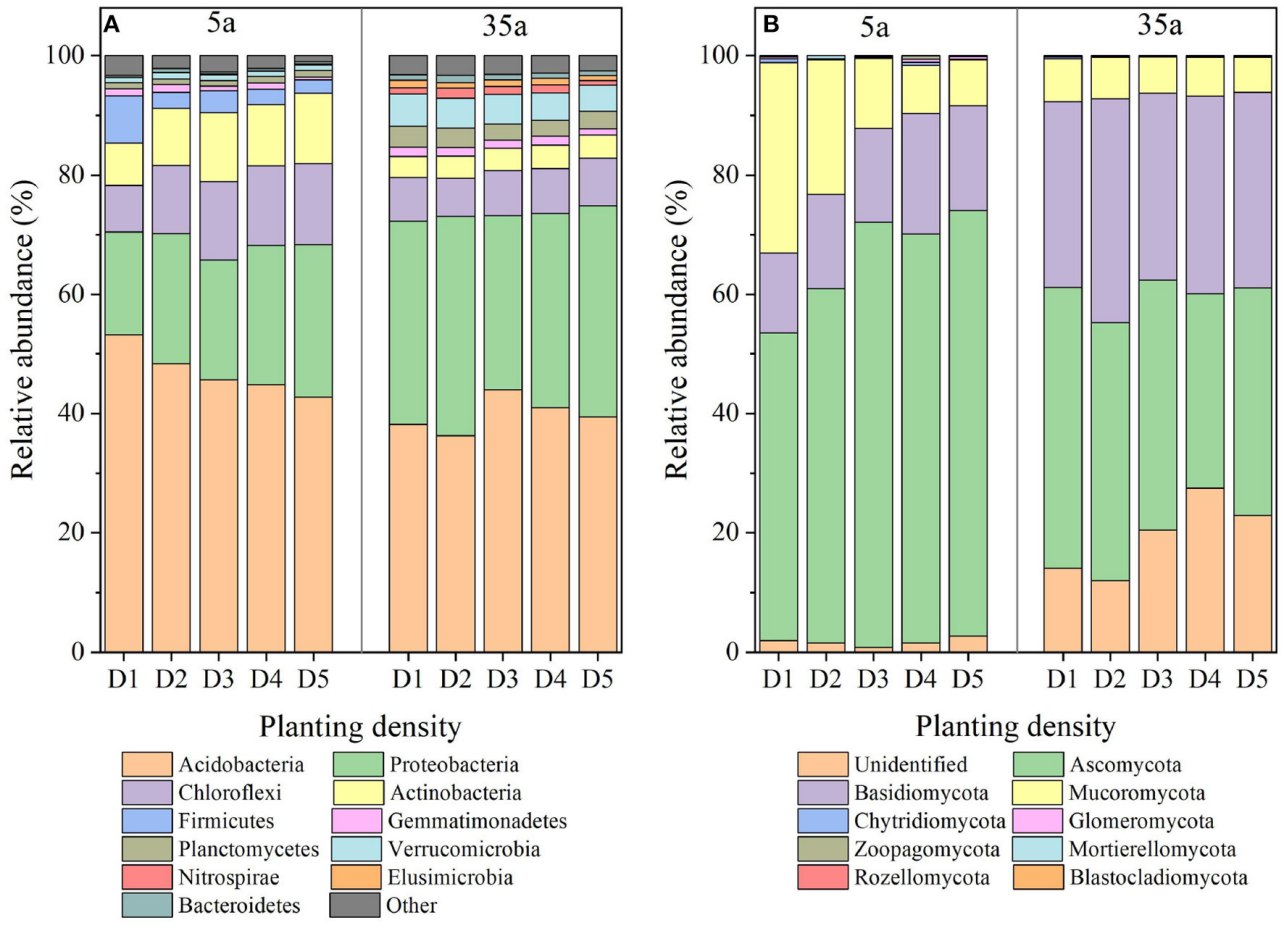
Bacterial **(A)** and fungal **(B)** community patterns in 5-year-old (left side) and 35-year-old (right side) plantations. D1, 1,667 stems ha^−1^; D2, 3,333 stems ha^−1^; D3, 5,000 stems ha^−1^; D4, 6,667 stems ha^−1^; D5, 10,000 stems ha^−1^.

The dominant bacterial phyla (relative abundance > 1%) were as follows: Acidobacteria, Proteobacteria, Chloroflexi, Actinobacteria, Firmicutes, Gemmatimonadetes, Planctomycetes, and Verrucomicrobia at age 5 and Acidobacteria, Proteobacteria, Chloroflexi, Verrucomicrobia, Actinobacteria, Planctomycetes, Gemmatimonadetes, Nitrospirae, Elusimicrobia, and Bacteroidetes at age 35 (Figure 3A). The relative abundance of Acidobacteria, Firmicutes, and Gemmatimonadetes decreased with planting density at age 5, while Chloroflexi and Actinobacteria increased (Supplementary Table 2). No differences in the relative abundance of these phyla among five planting densities were observed at age 35.

The dominant fungal phyla at age 5 included Ascomycota, Basidiomycota, and Mucoromycota, with average contributions of 51.7–71.4, 13.3–20.1, and 7.6–31.9%, respectively (Figure 3B, left side). The relative abundance of Ascomycota and Basidiomycota increased with planting density at age 5 while Mucoromycota decreased (Supplementary Table 3). At age 35, the dominant fungal phyla included Ascomycota, Basidiomycota, and Mucoromycota with average contributions of 32.6–47.1, 31.1–37.5, and 5.9–7.2%, respectively (Figure 3B, right side). The relative abundance of Ascomycota in D4 was lower than that in D1, D2, and D3, and it was higher in D1 and D2 than in D5 (Supplementary Table 3). The relative abundance of Basidiomycota in D2 was higher than that in the other four planting densities (Supplementary Table 3).

### Relationships Among Soil Physicochemical Properties, Enzyme Activities, Microbial Diversity, and Microbial Dominant Phyla Abundance

**Figure 4:**
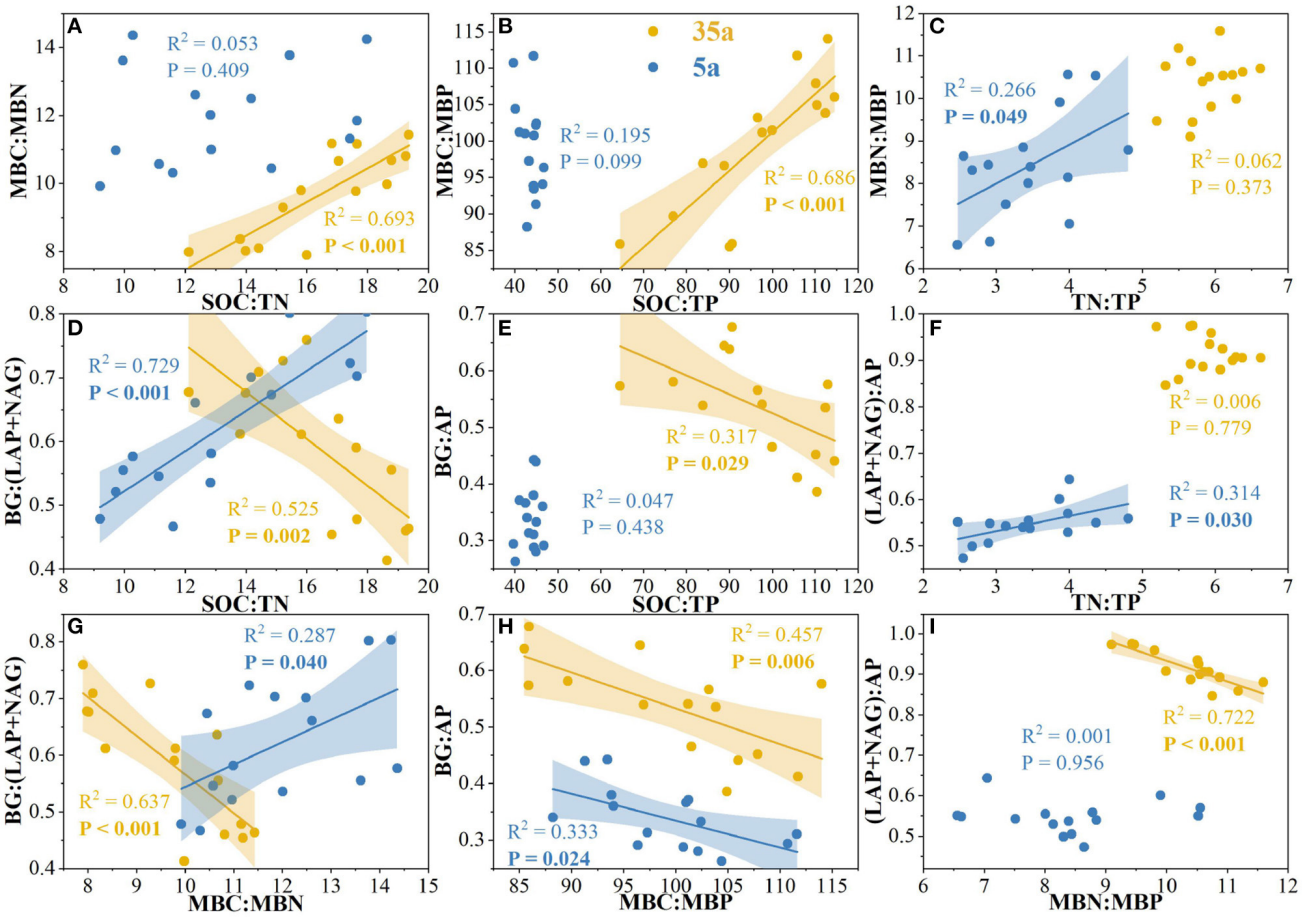
Relationships among soil carbon (C)/nitrogen (N)/phosphorus (P) stoichiometry, microbial biomass C/N/P stoichiometry, and enzymatic C/N/P stoichiometry in 5-year-old (5a, blue) and 35-year-old (35a, yellow) plantations. C acquisition is represented by β-glucosidase (BG); N acquisition is measured by the potential activities of leucine aminopeptidase (LAP) and β-1,4-N-acetylglucosaminidase (NAG); P acquisition is measured as acid phosphatase (AP) activity.

**Figure 5:**
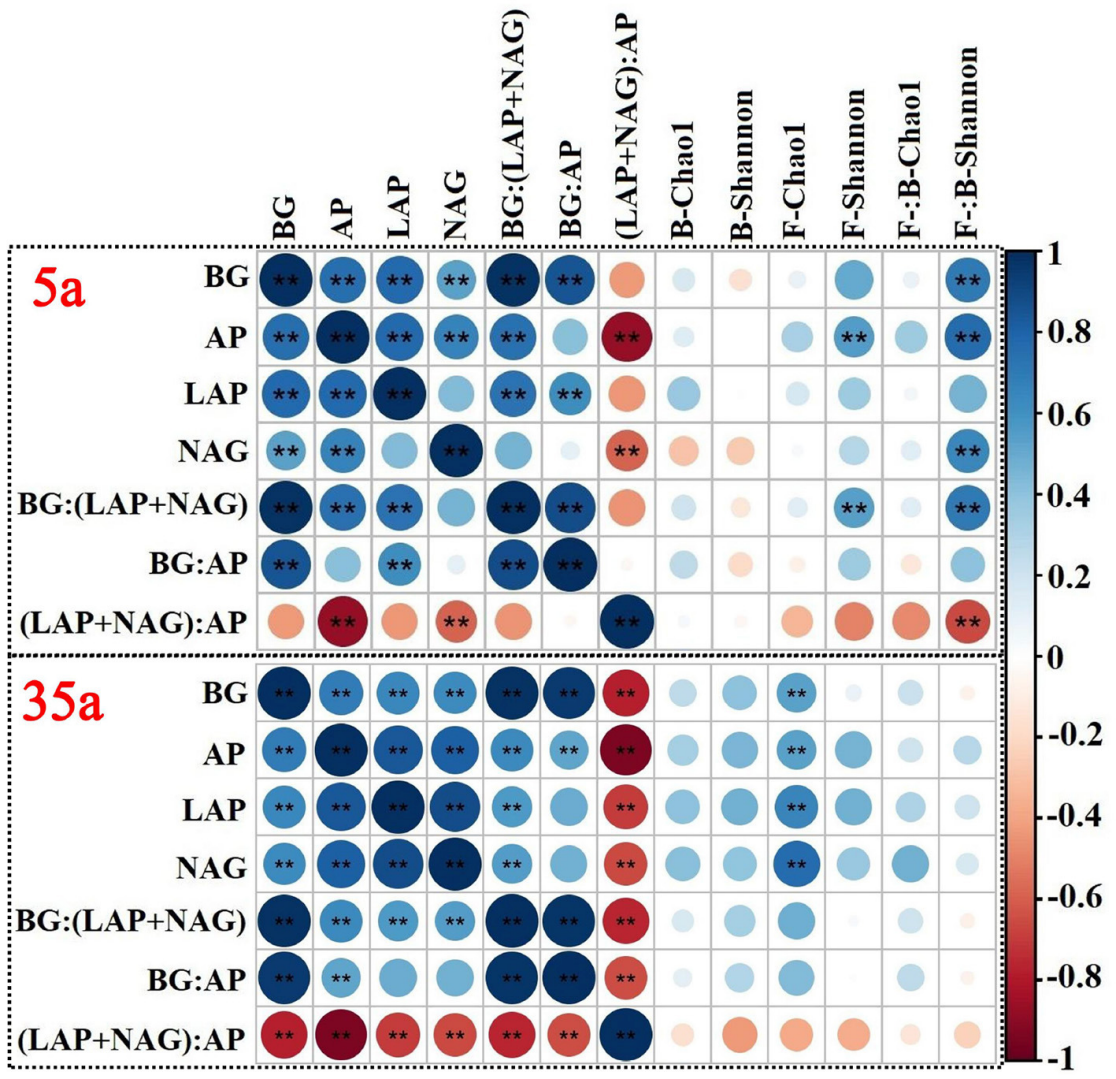
Spearman's rank correlation coefficients between enzyme activity and microbial alpha diversity (Chao1 and Shannon) in 5-year-old (top side) and 35-year-old (bottom side) plantations. BG, β-glucosidase; LAP, leucine aminopeptidase, NAG, β-1,4-N-acetylglucosaminidase; AP, acid phosphatase; B, bacteria; F, fungi.

At age 5, significant correlations were observed between TN/TP and MBN/MBP, between SOC/TN and BG/(LAP+NAG), between TN/TP and (LAP+NAG)/AP, between MBC/MBN and BG/(LAP+NAG), and between MBC/MBP and BG/AP (Figures 4C,D,F–H). The activities of BG, AP, and NAG were positively related to the ratio of fungal to bacterial Shannon index; moreover, positive relationships between AP and fungal Shannon index were observed at age 5 (Figure 5). At age 35, positive relationships between SOC/TN and MBC/MBN and between SOC/TP and MBC/MBP were found (Figures 4A,B). Negative relationships were found between SOC/TN and BG/(LAP+NAG), between SOC/TP and BG/AP, between MBC/MBN and BG/(LAP+NAG), between MBC/MBP and BG/AP, and between MBN/MBP and (LAP+NAG)/AP (Figures 4D,E,G–I). Moreover, BG, AP, LAP, and NAG were positively related to the fungal Chao1 index (Figure 5).

At age 5, microbial dominant phyla abundances were mainly affected by SM, TN, TP, SOC/TN, and TN/TP; moreover, enzyme activities were strongly affected by the abundance of bacterial Acidobacteria, Chloroflexi, and Gemmatimonadetes and of fungal Ascomycota and Mucoromycota (Supplementary Figure 2, left side). In contrast, microbial dominant phyla abundances were mainly affected by pH, SOC, and SOC/TP; moreover, enzyme activities were strongly affected by the abundance of bacterial Chloroflexi and Planctomycetes and fungal Ascomycota (Supplementary Figure 2, right side).

### Soil Physicochemical Factors and Microbial Biomass Controls on Enzyme Activity and Microbial Diversity

**Figure 6:**
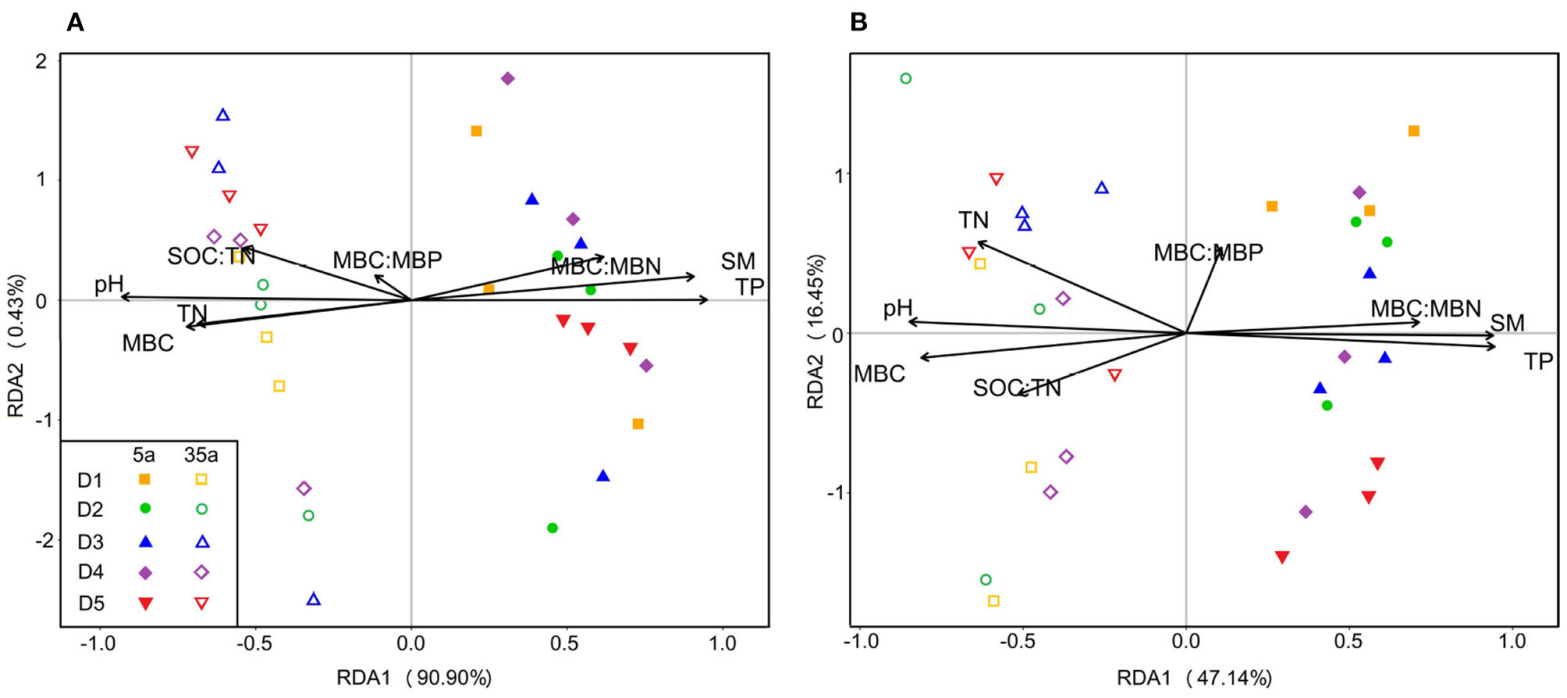
Ordination plots of the results from the redundancy analysis (RDA) to identify the relationships between bacterial **(A)** or fungal **(B)** communities and microbial biomass and soil physicochemical properties (black arrows) in 5-year-old (closed symbols) and 35-year-old (open symbols) plantations. BG, β-glucosidase; LAP, leucine aminopeptidase, NAG, β-1,4-N-acetylglucosaminidase; AP, acid phosphatase; MBC, microbial biomass carbon; MBN, microbial biomass nitrogen; MBP, microbial biomass phosphorus; SM, soil moisture; SOC, soil organic carbon; TN, total nitrogen; TP, total phosphorus; B, bacteria; F, fungi.

Redundancy analysis (RDA) demonstrated that soil biotic and abiotic factors together accounted for 91.33 and 63.59% of the variation in bacterial (Figure 6A) and fungal communities (Figure 6B), respectively. RDA results revealed that strong positive relationships between microbial communities and SM, TP, and MBC/MBN and negative relationships between microbial communities and pH, TN, SOC/TN, and MBC were observed at age 5, while the opposite patterns were observed at age 35 (Figure 6, Supplementary Table 4).

## Discussion

### Stoichiometric Imbalances With Planting Density and Stand Age

Our results demonstrated that soil, microbial, and enzymatic C/N/P stoichiometry differed among planting densities with the development of plantations from 5 to 35 years (Figure 2), thereby supporting our first hypothesis that there are stoichiometric imbalances between microbial biomass and their demand for nutrients with planting density during the development of plantations from 5 to 35 years. Enzymatic C/N and C/P ratios increased with planting density at age 5; moreover, enzymatic N/P ratio decreased with planting (Figures 2G–I). The opposite pattern was observed at age 35. This demonstrates that soil microorganisms provide more resources and energy for soil N-acquiring enzyme production other than P-acquiring enzymes with planting density during the development of plantations, indicating the shift from P- to N-limitation for microorganisms from 5 to 35 years. This was supported by the N-limitation of Chinese fir plantations after 12–22 years of development (Tong et al., 2020). Therefore, our first hypothesis regarding the P-limitation on microorganisms with planting density and stand age was only supported at age 5.

Soil eco-enzymatic stoichiometry was affected by plant residues since variations in plant tissue stoichiometry (Martiny et al., 2006). Therefore, the N-limitation could be attributed to increased input of coniferous litters with a higher C/N ratio stocks in high-density plantations (Table 1; Shao et al., 2017; Tong et al., 2021). The microbial growth rate is generally higher in soils with a lower soil C/N ratio, and microorganisms would release less C through respiration (Zhao et al., 2018). Conversely, microorganisms would convert more C to their biomass in soils with a higher C/N ratio (Schimel, 2016; Shoemaker et al., 2017). Importantly, this is supported by the negative correlation between MBC/MBN and BG/(LAP+NAG) with a planting density at age 35 (Figure 4). Another factor of N-limitation of microorganisms is that enzyme production itself requires investment N (Schimel and Weintraub, 2003). Moreover, the C/N ratio of proteins is usually much lower than the C/N of microbial biomass (Schimel and Weintraub, 2003). In addition, the greater amounts of fine root biomass in high-density plantations (Table 1) likely provided more resources to microorganisms, by increasing soil C contents (Creamer et al., 2014; Zhao et al., 2018). Accordingly, to acclimate to the N-limitation, microorganisms tend to secrete more N-acquiring enzymes than C-acquiring enzymes (Sinsabaugh et al., 2013; Rosinger et al., 2019; Li et al., 2020), thereby resulting in a decrease in enzymatic C/N and an increase in enzymatic N/P with planting density during the development of plantations from 5 to 35 years (Figure 2).

### Responses of Microorganisms to Stoichiometric Imbalances

Microbial biomass and enzyme activity increased with planting density in 5-year-old plantations, while the opposite pattern was observed in 35-year-old plantations (Figure 1, Table 1). Therefore, the second hypothesis that microbial biomass and enzyme activity would decrease with planting density was only supported at age 35. These results are in line with the findings reported from *C. lanceolata* plantations (Wu et al., 2011; Wang et al., 2018, 2019) and *Platycladus orientalis* plantations (Wang and Shi, 2016). Therefore, microbial growth and activity were limited by increasing planting density with the development of plantations. The microbial metabolism theory (Sinsabaugh et al., 2008) proposed that soil microorganisms adopted a survival strategy that is increasing enzyme activity in the sufficient resource and decreasing enzyme activity in the deficient resource. At age 5, the intensity of competition between microorganisms and plants was weak, and the bioavailability of soil nutrients to microorganisms was relatively high. Moreover, fine root biomass increased with planting density (Table 1). Accordingly, the microbial enzymatic synthesis capacity was strong, which ultimately promoted soil enzyme activity (Li et al., 2020). By contrast, at age 35, plants grew with large roots and occupied an absolute competitive advantage (Yang et al., 2020), and the depletion of nutrients (especially N and P) increased with planting density (Wang et al., 2021b). Therefore, the availability of nutrients cannot meet microbial demands, which ultimately limits soil enzyme activity.

Soil enzyme activity is mainly affected by microorganisms and plant roots (Gu et al., 2009). Increased root biomass is expected to stimulate root exudates into soils, promoting microbial activity (Castorena et al., 2016; Fraser et al., 2017). If so, high planting densities with higher fine root biomass would increase enzyme activity. Interestingly, the result was the opposite in 35-year-old plantations, suggesting that soil microorganisms contributed much more to enzyme activity than plant roots. The decrease in enzyme activity with planting density in 35-year-old plantations could be partially by decreasing biomass of understory vegetation (Table 1; Rodriguez-Loinaz et al., 2008; Wang et al., 2021a). As the main driving factor for plant litter decomposition, soil enzymes produced by microorganisms strongly depend on the species and stoichiometry of the plant litter (Cleveland and Liptzin, 2007; Baker and Allison, 2017; Rosinger et al., 2019). From this perspective, soil microbial biomass and enzyme activity should be higher in plantations with a higher amount and diversity of understory vegetation. Importantly, such a pattern was observed in 35-year-old plantations.

Negative relationships between MBC/MBN and BG/(LAP+NAG), between MBC/MBP and BG/AP, and between MBN/MBP and (LAP+NAG)/AP (Figure 4) supported the third hypothesis. Soil C/N and C/P ratios were positively correlated with MBC/MBN and MBC/MBP ratios at age 35 (Figure 4), indicating that microorganisms adjusted their biomass C/N/P ratios to meet the nutrient stoichiometry of their substrates with the development of plantations (Mooshammer et al., 2014). This can be explained by the central concept of stoichiometric invariance (homeostasis) in ecological stoichiometry (Sterner and Elser, 2002). This concept proposed that non-homeostatic organisms would change their biomass C/N/P in response to the variation in soil C/N/P ratios with two potential mechanisms: (i) microbial storage of excess elements, resulting in a convergence between the biomass and resource stoichiometries, and (ii) changes in microbial community structure, resulting in shifts in biomass stoichiometry (Mooshammer et al., 2014). Essentially, the first mechanism seems to be confirmed by positive relationships between SOC/TN and MBC/MBN and between SOC/TP and MBC/MBP at age 35 (Figure 4), indicating a physiological adjustment of non-homeostatic microorganisms' stoichiometry. Moreover, our studies demonstrated changes in microbial community composition, and the ratio of fungal to bacterial diversity increased with planting density and stand age (Figure 3, Table 2). Accordingly, microorganisms may adopt non-homeostatic behaviors to reduce the stoichiometric imbalance to their resources with planting density during the development of plantations from 5 to 35 years.

We found that changes in soil bacterial and fungal compositions were mainly dependent on limited nutrients as observed in microbial community abundances positively related to TP at age 5 but related to TN and SOC/TN at age 35 (Supplementary Table 4). This again demonstrates the shift from P- to N-limitation for microorganisms from 5 to 35 years. To cope with the shift from P- to N-limitation, microorganisms changed the relative abundance of dominant phyla. For example, Proteobacteria, fast-growing and dominant bacteria, require higher P to synthesize a large amount of RNA to achieve a high rate of cell division (Elser et al., 2003; El Zahar et al., 2008). Moreover, the compositions of Proteobacteria, such as Rhizobiales, are considered to be rhizosphere plant-promoting bacteria (Jorquera et al., 2014) and can fix atmospheric nitrogen in symbiosis with plants (Van der Heijden et al., 2008). The same pattern was observed for Nitrospirae that can promote nitrogen accumulation (DeLuca et al., 2002). Thus, such an increase in the abundance of Proteobacteria and Nitrospirae should be an effective way to cope with the shift from P- to N-limitation of microorganisms with stand age. Remarkably, the fungal-to-bacterial ratios of either Chao1 (0.15–0.19 and 0.96–1.10 at age 5 and 35, respectively), or Shannon (0.63–0.71 and 1.20–1.41) index increased with stand age (Table 2). This was supported by the observed decreased fungal-to-bacterial ratio with inorganic nitrogen fertilization (Bloem and Vos, 2004). As compared to bacteria, the ability of fungi to degrade more recalcitrant litter, such as cellulose and lignin, in coniferous forests is stronger (Lauber et al., 2008). Moreover, the coniferous litter with a higher C/N ratio was increased with planting density and stand age (Table 1). Thus, an increased fungal-to-bacterial ratio may be another important pathway to cope with the shift from P- to N-limitation of microorganisms.

Finally, no significant differences in the relative abundance of the predominant bacteria were found among different planting densities at age 35, suggesting that bacterial communities strongly resist the change in planting density. This is supported by the fact that bacterial communities do not respond to plant–soil feedback, while the responses of fungal communities to plant–soil feedback are more remarkable ((Kardol et al., 2007; Lorenzo et al., 2010)). This is mainly because bacterial communities have a small-scale niche in the soil and are less symbiotic with the plant as compared to fungal communities (Sun et al., 2017). Therefore, the responses of bacterial communities to soil property changes caused directly or indirectly by planting density are weaker.

## Conclusion

Microbial biomass, microbial diversity, and C-, N-, and P-acquiring enzyme activities increased with planting density at age 5, but the opposite pattern was observed at age 35. Contrasting patterns of stoichiometric imbalances between soil microorganisms and their demand for nutrients with planting density at ages 5 and 35 indicated changes in microbial nutrient limitation. Moreover, our study demonstrated that soil microbial nutrient limitation shifted from P- to N-limitation with planting density during the development of plantations from 5 to 35 years. Soil microorganisms may cope with the imbalance of stoichiometry by: (i) coordinating the adjustment of the stoichiometry of microorganisms and enzymes; (ii) changing the relative abundance of dominant microbial phyla; and (iii) increasing the fungal-to-bacterial diversity ratio. These findings support the ecological theory that microorganisms optimally allocate their resources when resources are limited. Altogether, our findings highlight that (i) stoichiometric imbalances are the main driver in shaping soil microbial communities and (ii) enzymatic stoichiometry can identify and explain microbial nutrient limitations and provide deeper insights for predicting nutrient bioavailability to microorganisms and modeling microbially mediated nutrient cycling in terrestrial ecosystems.

## Data Availability Statement

The datasets presented in this study can be found in online repositories. The names of the repository/repositories and accession number(s) can be found at: NCBI GenBank; PRJNA811461.

## Author Contributions

CW: conceptualization, methodology, validation, formal analysis, data curation, writing-original draft preparation, writing-review and editing, and visualization. RJ: conceptualization, methodology, validation, formal analysis, resources, writing-review and editing, supervision, and funding acquisition. Both authors contributed to the article and approved the submitted version.

## Funding

This study was supported by the State Key Research Development Program of China (2016YFD0600302).

## Conflict of Interest

The authors declare that the research was conducted in the absence of any commercial or financial relationships that could be construed as a potential conflict of interest.

## Publisher's Note

All claims expressed in this article are solely those of the authors and do not necessarily represent those of their affiliated organizations, or those of the publisher, the editors and the reviewers. Any product that may be evaluated in this article, or claim that may be made by its manufacturer, is not guaranteed or endorsed by the publisher.
