# Supplementary material for: Adaptive Pathways of Microorganisms to Cope With the Shift From P- to N-Limitation in Subtropical Plantations
Source: Front Microbiol. 2022 Apr 29;13:870667. doi: 10.3389/fmicb.2022.870667 (PMC9100944; doi:10.3389/fmicb.2022.870667)
Supplement: Supplementary file 1 [file Data_Sheet_1.docx]

**Supplementary**

**
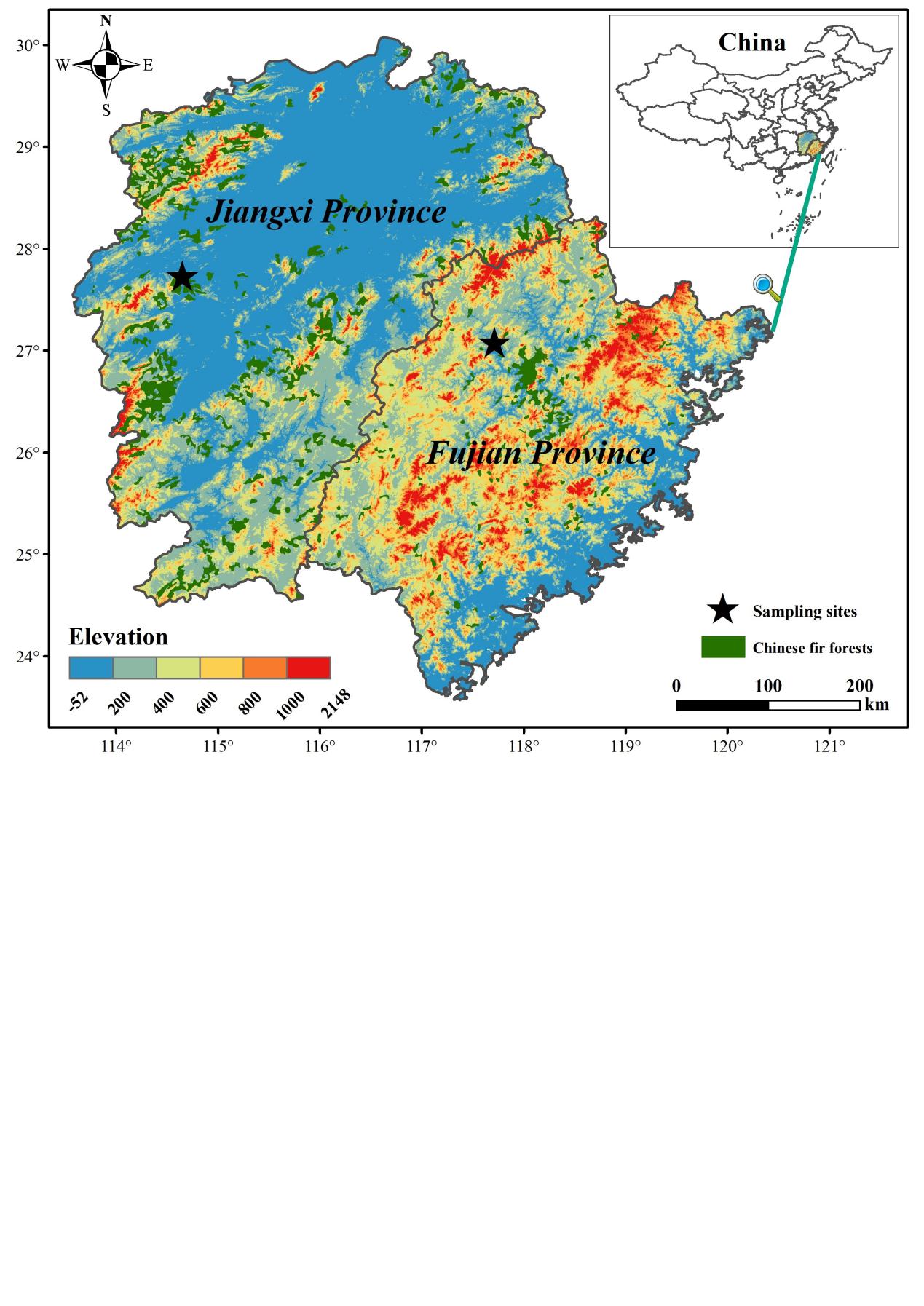
**

**Figure S1** Spatial extent of the study area. Two study sites have been labeled on the figure. The Chinese fir forests at Jiangxi Province and Fujian Province were from Compiling Committee of Vegetation Maps of 1:1000000 in China, obtained by National Cryosphere Desert Data Center (http://www.ncdc.ac.cn/).


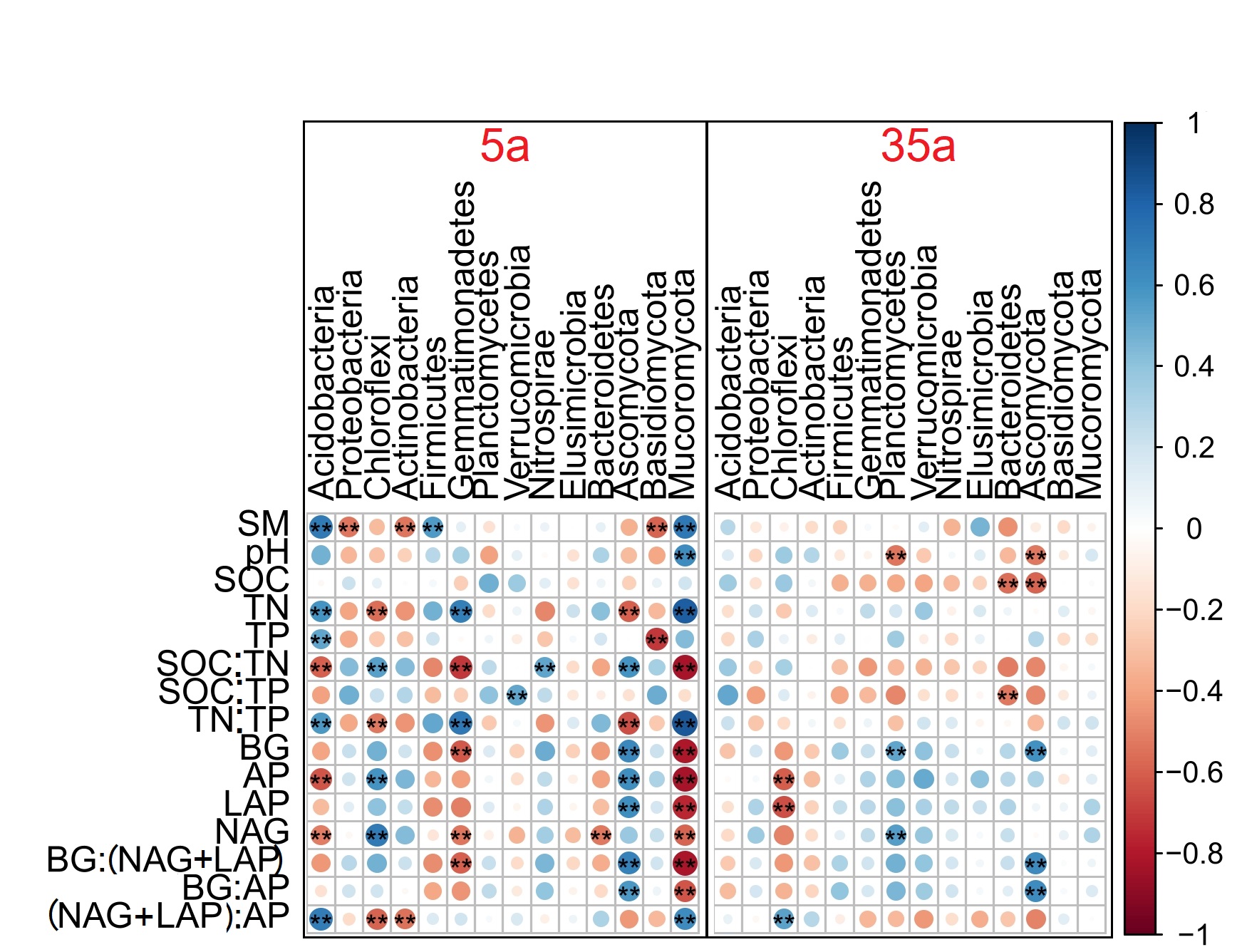


**Figure S2** Spearman’s rank correlation coefficients between microbial dominant phyla and enzyme activities and soil physicochemical properties in 5- (left side) and 35-year-old plantations (right side). Abbreviations: SM, soil moisture; SOC, soil organic carbon; TN, total nitrogen; TP, total phosphorus; BG, β-glucosidase; LAP, leucine aminopeptidase, NAG, β-1,4-N-acetylglucosaminidase; AP, acid phosphatase.

**Table S1** Statistics of bacterial and fungal high quality sequences and OTUs in 5- (5a) and 35-year-old (35a) plantations

|  |  | Bacterial | | Fungal | |
| --- | --- | --- | --- | --- | --- |
|  |  | Sequences | OTUs | Sequences | OTUs |
| 5a | D1 | 30527 ± 4292AB | 4533 ± 513A | 33973 ± 1008A | 952 ± 249AB |
|  | D2 | 27273 ± 2529B | 5057 ± 228A | 25632 ± 2345A | 790 ± 91B |
|  | D3 | 31531 ± 7346AB | 5021 ± 230A | 21678 ± 3088A | 988 ± 59AB |
|  | D4 | 38629 ± 5089A | 5526 ± 500A | 30379 ± 4895A | 1117 ± 233A |
|  | D5 | 30327 ± 3497AB | 4934 ± 166A | 27879 ± 1884A | 1215 ± 254A |
| 35a | D1 | 27230 ± 313a | 1700 ± 134a | 40020 ± 3568a | 2018 ± 112a |
|  | D2 | 25690 ± 1437a | 1737 ± 127a | 43586 ± 3594a | 1978 ± 121a |
|  | D3 | 27647 ± 677a | 1717 ± 123a | 33336 ± 2703ab | 1842 ± 201ab |
|  | D4 | 20671 ± 4234b | 1691 ± 251a | 30771 ± 2802b | 1572 ± 139b |
|  | D5 | 26214 ± 995a | 1649 ± 159a | 38227 ± 6220ab | 1769 ± 219ab |

The data are shown as the means ± standard deviations (n = 3). Different uppercase and lowercase letters represent significant differences among planting densities at age 5 and 35, respectively. Abbreviations: D1, 1667 stems ha^–1^; D2, 3333 stems ha^–1^; D3, 5000 stems ha^–1^; D4, 6667 stems ha^–1^; D5, 10000 stems ha^–1^.

**Table S2** The relative abundance of bacterial and fungal dominant phyla in 5- (5a) and 35-year-old (35a) Chinese fir plantations of different stand densities

|  | 5a | | | | |  | 35a | | | | |
| --- | --- | --- | --- | --- | --- | --- | --- | --- | --- | --- | --- |
|  | D1 | D2 | D3 | D4 | D5 |  | D1 | D2 | D3 | D4 | D5 |
| Acidobacteria | 53.27 ± 2.34a | 48.31 ± 1.92ab | 45.64 ± 8.52ab | 44.82 ± 0.96ab | 42.76 ± 3.10b |  | 38.19 ± 3.27a | 36.29 ± 3.16a | 44.00 ± 8.32a | 41.00 ± 2.96a | 39.42 ± 4.52a |
| Proteobacteria | 17.22 ± 4.52a | 21.91 ± 0.89a | 20.11 ± 5.86a | 23.44 ± 3.59a | 25.64 ± 2.52a |  | 34.10 ± 3.81a | 36.80 ± 1.63a | 29.23 ± 4.27a | 32.56 ± 3.52a | 35.44 ± 4.97a |
| Chloroflexi | 7.78 ± 1.22b | 11.37 ± 0.52ab | 13.20 ± 2.40b | 13.29 ± 4.66b | 13.55 ± 1.36b |  | 7.28 ± 0.22a | 6.40 ± 0.34a | 7.52 ± 1.37a | 7.51 ± 0.59a | 7.99 ± 0.44a |
| Actinobacteria | 7.06 ± 1.13b | 9.57 ± 1.21ab | 11.51 ± 2.90a | 10.28 ± 1.86ab | 11.75 ± 0.86a |  | 3.54 ± 0.34a | 3.71 ± 0.39a | 3.75 ± 0.49a | 3.95 ± 1.04a | 3.89 ± 0.47a |
| Firmicutes | 7.91 ± 4.08a | 2.71 ± 0.22b | 3.65 ± 1.86ab | 2.54 ± 0.17b | 2.26 ± 0.11b |  | 0.04 ± 0.01a | 0.05 ± 0.02a | 0.03 ± 0.01a | 0.06 ± 0.02a | 0.04 ± 0.02a |
| Gemmatimonadetes | 1.19 ± 0.24a | 1.29 ± 0.16a | 0.81 ± 0.25ab | 1.07 ± 0.55ab | 0.46 ± 0.15b |  | 1.58 ± 0.34a | 1.42 ± 0.32a | 1.39 ± 0.23a | 1.48 ± 0.26a | 1.02 ±0.24a |
| Planctomycetes | 1.05 ± 0.23a | 0.93 ± 0.14a | 0.89 ± 0.08a | 1.08 ± 0.10a | 1.06 ± 0.12a |  | 3.50 ± 0.48a | 3.30 ± 0.73a | 2.65 ±0.53a | 2.63 ± 0.29a | 2.95 ±0.33a |
| Verrucomicrobia | 0.83 ± 0.33a | 1.07 ± 0.25a | 0.98 ± 0.10a | 0.86 ± 0.06a | 0.94 ± 0.11a |  | 5.38 ± 0.60a | 4.95 ± 0.64a | 4.96 ± 0.77a | 4.69 ± 0.42a | 4.31 ± 0.33a |
| Nitrospirae | 0.03 ± 0.03a | 0.06 ± 0.02a | 0.14 ± 0.01a | 0.05 ± 0.01a | 0.18 ± 0.09a |  | 1.09 ± 0.25a | 1.70 ± 0.26a | 1.36 ± 0.14a | 1.30 ± 0.55a | 0.76 ± 0.25a |
| Elusimicrobia | 0.03 ± 0.01a | 0.01 ± 0.01a | 0.01 ± 0.01a | 0.02 ± 0.01a | 0.01 ± 0.01a |  | 1.23 ± 0.18a | 0.94 ± 0.23a | 1.11 ± 0.13a | 1.12 ± 0.29a | 0.92 ± 0.31a |
| Bacteroidetes | 0.32 ± 0.10a | 0.60 ± 0.23a | 0.33 ± 0.08a | 0.39 ± 0.19a | 0.32 ± 0.16a |  | 0.88 ± 0.20a | 1.26 ± 0.24a | 0.92 ± 0.19a | 0.84 ± 0.24a | 0.76 ± 0.15a |

The data are shown as the means ± standard deviations (n = 3). Different lowercase letters represent significant differences among planting densities in the same age plantations. Different lowercase letters represent significant differences between different planting densities (*p* < 0.05). Abbreviations: D1, 1667 stems ha^–1^; D2, 3333 stems ha^–1^; D3, 5000 stems ha^–1^; D4, 6667 stems ha^–1^; D5, 10000 stems ha^–1^.

**Table S3** The relative abundance of fungal dominant phyla in 5- (5a) and 35-year-old (35a) Chinese fir plantations of different stand densities

|  |  | Ascomycota | Basidiomycota | Mucoromycota |
| --- | --- | --- | --- | --- |
| 5a | D1 | 51.70 ± 7.36b | 13.31 ± 1.31b | 31.92 ± 6.04a |
|  | D2 | 59.46 ± 6.74ab | 15.76 ± 3.12ab | 22.54 ± 2.49b |
|  | D3 | 71.31 ± 8.39a | 15.66 ± 7.23ab | 11.70 ± 0.86c |
|  | D4 | 68.57 ± 3.28a | 20.14 ± 0.48a | 8.10 ± 2.61c |
|  | D5 | 71.37 ± 1.03a | 17.53 ± 1.21a | 7.63 ± 1.15c |
| 35a | D1 | 47.14 ± 7.71a | 31.12 ± 11.71b | 7.18 ± 0.15a |
|  | D2 | 43.35 ± 11.47a | 37.50 ± 10.90a | 6.94 ± 0.98a |
|  | D3 | 42.00 ± 3.39ab | 31.28 ± 9.06b | 6.04 ± 0.16a |
|  | D4 | 32.64 ± 8.45c | 33.03 ± 7.34b | 6.58 ± 2.19a |
|  | D5 | 38.31 ± 9.67bc | 32.71 ± 10.14b | 5.88 ± 1.45a |

The data are shown as the means ± standard deviations (n = 3). Different lowercase letters represent significant differences among planting densities in the same age plantations. Different lowercase letters represent significant differences between different planting densities (*p* < 0.05). Abbreviations: D1, 1667 stems ha^–1^; D2, 3333 stems ha^–1^; D3, 5000 stems ha^–1^; D4, 6667 stems ha^–1^; D5, 10000 stems ha^–1^.

**Table S4** The permutation test of soil properties of different planting densities in 5- and 35-year-old plantations based on soil bacterial and fungal communities. The P values were based on 999 permutations.

|  | Bacterial community | | | |  | Fungal community | | | |
| --- | --- | --- | --- | --- | --- | --- | --- | --- | --- |
|  | RDA1 | RDA2 | r^2^ | P |  | RDA1 | RDA2 | r^2^ | P |
| SM | 0.990 | 0.142 | 0.831 | **0.001** |  | 0.999 | -0.006 | 0.834 | **0.001** |
| pH | -0.996 | -0.086 | 0.826 | **0.001** |  | -0.999 | 0.030 | 0.680 | **0.001** |
| TN | -0.988 | -0.157 | 0.486 | **0.001** |  | -0.954 | 0.298 | 0.491 | **0.001** |
| TP | 0.996 | 0.093 | 0.867 | **0.001** |  | 0.999 | -0.032 | 0.845 | **0.001** |
| MBC | -0.987 | -0.161 | 0.540 | **0.001** |  | -0.998 | -0.065 | 0.630 | **0.001** |
| SOC:TN | -0.993 | 0.114 | 0.278 | **0.008** |  | -0.968 | -0.251 | 0.299 | **0.010** |
| MBC:MBN | 0.976 | 0.218 | 0.435 | **0.001** |  | 0.999 | 0.032 | 0.482 | **0.001** |
| MBC:MBP | -0.927 | 0.376 | 0.019 | 0.766 |  | 0.498 | 0.867 | 0.105 | 0.248 |

Note: RDA1 and RDA2 represent the first and second RDA axes, respectively. Abbreviations: SM, soil moisture; SOC, soil organic carbon; TN, total nitrogen; TP, total phosphorus; MBC, microbial biomass carbon; MBN, microbial biomass nitrogen; MBP, microbial biomass phosphorus.
